# Cardiac Anatomic Considerations in Pediatric Electrophysiology

**Published:** 2008-05-01

**Authors:** Samuel J Asirvatham

**Affiliations:** Division of Cardiovascular Diseases, Department of Internal Medicine, Mayo Clinic, Rochester, Minnesota

**Keywords:** cardiac anatomy, conduction system, electrophysiology, children, radiofrequency ablation

## Abstract

Although interventional electrophysiology and the use of radiofrequency energy to cure various arrhythmias primarily developed in the adult population, similar applications in children have grown dramatically over the last decade. The anatomic basis for various arrhythmias is critically important for the pediatric ablationists to appreciate. Such understanding allows the use of alternative technique to affect cure while avoiding complications. Further, because of the relatively small heart and less thick myocardium in children, without the appreciation of the underlying cardiac anatomic relationships, collateral injury, for example to the arterial system, may occur. In this review, the cardiac anatomic consideration important in approaching various supraventricular and ventricular arrhythmias in the normal heart is discussed.

## Introduction

The last two decades have witnessed tremendous change in the care of children with symptomatic cardiac arrhythmia. The anatomic basis for various cardiac arrhythmias (in children and adults) has been much better understood, particularly for common supraventricular tachyarrhythmia.

While cardiac electrophysiology still forms the basis for electrophysiological study and radiofrequency ablation for supraventricular arrhythmia, pure anatomic approaches are evolving with appropriate adjunctive electrophysiological maneuvers performed prior to energy delivery. Radiofrequency ablation for AV node reentry and paroxysmal atrial fibrillation is performed almost entirely as an anatomy-based ablation in many centers [1-2].

Radiofrequency ablation was initially reported as a feasible, successful, and safe therapy that could be used in children as an alternative to drug therapy in 1991 [3]. There are, however, significant challenges when performing the sometimes complex procedures in children, particularly the very young. An approach to some of these difficult scenarios encountered in pediatric cardiac electrophysiology is the subject of another manuscript in this series. In this paper, I will describe the anatomic basis for common arrhythmias with specific emphasis on variation in the pediatric population relevant to safe and affective ablation in these patients. We will confine ourselves to "normal" cardiac anatomy and the genesis of arrhythmia. In another paper found in this supplement, we will also cover an approach to the anatomy and ablation difficulty in children who had surgically corrected congenital heart disease.

## AV Node Reentrant Tachycardia (AVNRT)

Over 90% of supraventricular arrhythmia in children is a result of a reentrant mechanism [4]. The second most common reentrant supraventricular tachycardia (SVT) in children is AV node reentrant tachycardia. This arrhythmia is relatively less common in the very young and preadolescent child but becomes increasingly more prevalent in older children and teenagers. There are several reasons why an accurate understanding of the anatomic basis of this arrhythmia is important for pediatric electrophysiologists. These include the need to ablate in the vicinity of the normal conduction system in these patients with relatively small hearts (increasing the risk of heart block) and the need to distinguish this arrhythmia from other common arrhythmias in childhood including reentrant SVT using a septal bypass tract and adjunctional tachycardia.

### Anatomic Basis of AVNRT

**Figure 1:**
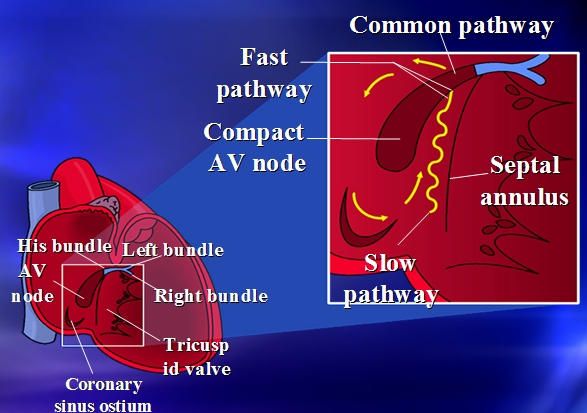
Illustration of the atrial input to the compact AV node. Posterior myocardium enters the AV node involving atrial myocardium of the coronary sinus and between the anterior rim of the coronary sinus and the tricuspid annulus. These fibers constitute the slow pathway, and when entering to the compact AV node, the propagated impulse travels simultaneously to the atrial myocardium via the anterior positioned fast pathway and through the lower AV node and His bundle to the ventricle. This simultaneous disbursement of the impulse gives rise to the very short R-P interval during AV node reentrant tachycardia.

In the initial few decades of cardiac electrophysiology, AVNRT was thought to represent a reentrant arrhythmia arising entirely from the compact AV node. This was because there was nearly simultaneous activation of the ventricle and atrium observed from the earliest recorded demonstration of this arrhythmia. If this were to have been the case (entire circuit in AV node), radiofrequency ablation for this arrhythmia without heart block would not have been possible. It later became recognized that AV node reentry could be reset and entrained from the atrium particularly the posteroseptal annular atrial tissue (Figure 1). Importantly, resetting of the tachycardia could be demonstrated *without* necessarily effecting the His bundle electrogram, yet either advancing or delaying the subsequent atrial electrogram [2]. Pioneering work such as this gave rise to the notion that ablation in the atrium could potentially represent a definitive treatment for this arrhythmia as has turned out to be the case.

The circuit for this arrhythmia, however, is far from completely understood. While it is accepted that atrial inputs to the AV node are necessary components to the circuit (see below), certain less commonly observed phenomena do suggest a distinct difference from AVNRT and any other macroreentrant atrial tachycardia. AVNRT can be dissociated from the ventricle either with block occurring in an infra-Hisian location (more common) or above the bundle of His. Also clearly observed and well documented are cases of AV node reentry with dissociation of the tachycardia from all recorded atrial tissue (upper common pathway block, see below).

### What is the Circuit for AVNRT?

The sinus impulse originates epicardially at the junction of the crista terminalis and the superior vena cava, where as the atrioventricular conduction system is located anteriorly and septally on the annulus. Specifically, the penetrating bundle of His is consistently found at the junction of the noncoronary cusp and right coronary cusp of the aortic valve where it meets the commissure of the anterior and septal leaflets of the tricuspid valve (membranous intraventricular septum). The compact AV node itself is located relatively more atrial and more posteriorly (closer to the coronary sinus) in the mid-septal tricuspid annulus when compared to the penetrating bundle of His. Although the compact AV node is relatively atrial to the His bundle, it is always found ventricular to the Eustachian ridge and the underlying tendon of Todaro within the so-called triangle of Koch. This triangle is bounded anteriorly by the tricuspid annulus, posteriorly by the tendon of Todaro, and inferiorly by the coronary sinus.

The atrial inputs into the AV node that carry the sinus node impulse (or paced impulse) are not diffuse but along distinct anatomic pathways. This is because of the anatomic obstacles in the atrium (particularly right atrium) that the impulse must circumvent to reach the AV node. The atrial myocardial input to the AV node that occurs superior to the fossa ovalis and through the apex of the triangle of Koch is referred to as the fast pathway. The posterior inputs to the AV node traverse to this structure along the interatrial septum. These posterior inputs may involve the myocardial sleeves along the coronary sinus and the posteroseptal right atrium in the region of the coronary sinus ostium. The posterior inputs are referred to as the slow pathway and may be more than one in number (right and left-sided slow pathways). The actual junction of these posterior inputs into the AV node is likely through extensions of compact AV nodal and transitional AV nodal tissue called the posterior horns. This anatomic distinction between the atrial myocardial inputs to the AV node forms the essential substrate for AV node reentrant tachycardia.

**Figure 2:**
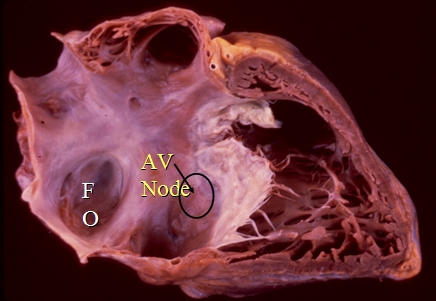
Autopsied heart dissected and shown in the right anterior oblique view (Right atrium and right ventricle). The fossa ovalis and coronary sinus constitute anatomic obstacles to the wave front from the high right atrium (sinus node, pacing) to reach the compact AV node. Transmission anterior and superior to the fossa ovalis anatomically constitutes the fast pathway input to the AV node, whereas the propagated wave front posterior and ventricular to the fossa ovalis in the region of the coronary sinus constitutes the anatomic slow pathway (see text for details).

It should be noted that the distinction between the fast and slow pathways, while although partially reflective of the different conduction and refractory properties, is fundamentally an anatomic distinction (Figure 2). In other words, regardless of conduction time and H-A interval, anterior inputs to the AV node constitute the *fast* pathway.

This anatomic distinction of the fast and slow pathway is not easily ascertained in the antegrade direction and involves complex entrainment maneuvers during AV node reentry to provide evidence. On the other hand, retrograde activation of the atrium during AV node reentry or ventricular pacing is straightforward in demonstrating proof of the anatomic separation of the two limbs of the AVNRT circuit. During typical AV node reentry or during ventricular pacing with retrograde activation of the fast pathway, the earliest recordable site of atrial activation is just behind the tendon of Todaro, close to the apex of the triangle of Koch. Whereas with atypical AV node reentry or retrograde activation via the slow pathway during ventricular pacing, the earliest recorded atrial electrograms are in the region of the coronary sinus ostium (posterior).

The fact that atrial myocardium is essential to the circuit of AV node reentry is more than semantics and forms the basis for all atrial AVNRT ablation procedures. While ablating either the anatomic fast or slow pathway will prevent future AVNRT, the exit site of the fast pathway is fairly close to the compact AV node and separated only from this structure by the tendon of Todaro. The slow pathway, however, (see below) can be ablated 4 of 5 cm from the compact AV node with little or no risk of AV block. This distinction in the anatomy of the two pathways to the AV node is critical for safe ablation in young children.

## Fast Pathway

It is important for ablationists to understand the fluoroscopic anatomy of the fast pathway. In the RAO projection as anticipated, the fast pathway site is more atrial (behind the Eustachian ridge) than the compact AV node and, of course, the penetrating bundle of His. The characteristic fluoroscopic appearance, however, when mapping the fast pathway, is best noted in the LAO projection (Figure 2). As the catheter is moved posteriorly (more atrially from the His location) with clockwise torque being applied on the catheter, there will be a sudden leftward movement of the catheter almost as if the fossa ovalis has been reached. This is because the Eustachian ridge keeps any catheter that is ventricular to the structure relatively rightward in the LAO projection, thus, a His bundle catheter (ventricular to the Eustachian ridge) will be more rightward than the fast pathway catheter (posterior to the Eustachian ridge). In typical AV node reentry, a catheter appropriately positioned in the fast pathway will record an earlier atrial electrogram than the atrial electrograms on the His bundle catheter itself.

## Anatomy of the Slow Pathway

**Figure 3:**
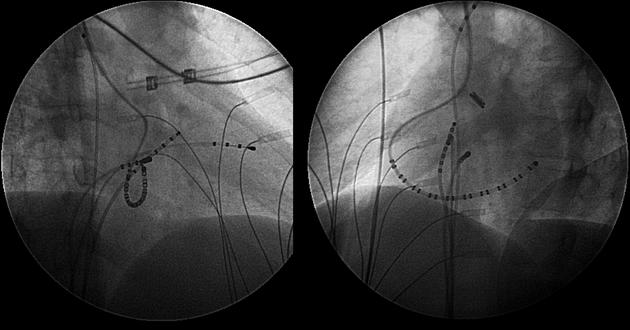
Fluoroscopic images illustrating the anatomic difference between the His bundle and the fast pathway. The left panel is the right anterior oblique (RAO) projection and the right panel is the left anterior oblique (LAO) projection. Note, in the LAO view, the quadrapolar ablation catheter points leftward when compared to the octapolar His bundle recording catheter placed on the septum. This is because the fast pathway being mapped by this catheter is behind the tendon of Todaro, allowing the catheter to point in a leftward manner. Accurate mapping of the fast pathway is important when ablating various supraventricular arrhythmias with early atrial activation on the atrial septum in children (see text for details).

The slow pathway fibers are funnel-like and triangular in section with the apex of the triangle inserting into the compact AV node and the base fanning out into the posterior interatrial septum both involving the fibers of the coronary sinus and the right posterior septal atrium (Figure 3). Thus, to ablate the slow pathway, either a single point ablation, relatively close to the compact AV node or a relatively long linear lesion at the level of the floor of the coronary sinus ostium is effective [5].

## Applications in Pediatric Electrophysiology

**Figure 4:**
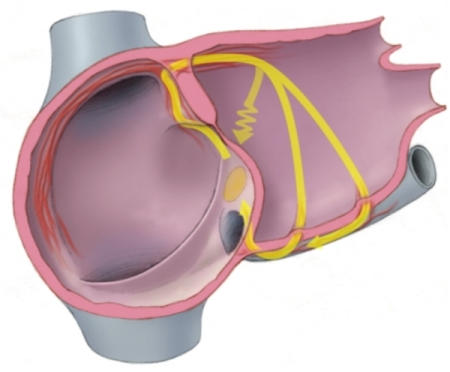
In children, at times there is anatomic continuity of the crista terminalis and the eustachian ridge in the right atrium. This anatomic fact paired with the relative rapid conduction times between the atria creates unusual coronary sinus activation patterns in some children with AV node reentry. The propagated impulse after exiting from the fast pathway may travel to the left atrium through Bachmann's bundle region and then enter the coronary sinus in its mid or distal portion through muscular connections and then travel back to the compact AV node region (slow pathway). In these instances, accurate mapping of the fast pathway will still show the earliest atrial activation, however, the coronary sinus activation may be eccentric.

1. Because of the relatively smaller hearts and thus closer proximity of the compact AV node to the atrial input sites, ablation in children should be performed as far away from the compact AV node as possible [6,7]. Understanding the anatomy of the AVNRT circuit, this can be effectively accomplished with a complete linear lesion at the level of the floor of the coronary sinus ostium. Because of the left-sided inputs to the AV node as well, ablation often is required slightly within the coronary sinus on the floor [8]. Care should be taken when ablating using this approach not to ablate on the roof of the coronary sinus near the ostium (compact AV node) and to avoid inadvertent cannulation of the middle cardiac vein. A catheter that has cannulated the middle cardiac vein is easily recognized when counter-clockwise torque is applied in the LAO projection. A catheter on the slow pathway region will immediately move rightward and away from the septum, but such movement is not possible and the tip will remain engaged when in the middle cardiac vein.
2. Because of relatively rapid conduction times in children, it can be difficult to distinguish whether retrograde activation is via the slow pathway or fast pathway. Thus, there is often simultaneous activation of the atrium as recorded on the His bundle catheter and the proximal coronary sinus catheter. Specific mapping, understanding the fluoroscopic anatomy as detailed above of the fast pathway will clarify the situation.
3. In children who have a persistent left superior vena cava, the coronary sinus including the ostium can be very large. This can make ablation of the slow pathway difficult. This difficulty is because of the lack of catheter stability created by the large coronary sinus ostium and the fact that multiple slow pathway inputs related to the diverse musculature of the enlarged coronary sinus will require attention. Again, linear ablation including careful ablation within the coronary sinus is most likely to be effective [9].
4. In children, because of relatively rapid conduction times through the fast pathway, typically AVNRT may present as a long R-P tachycardia. This is because in AVNRT, activation of the atria and ventricle occur from a common turnaround point likely within the compact AV node or next to it (slower common pathway). When fast pathway activation is rapid, then atrial activation proceeds ventricular activation, giving rise to a long R-P interval. The P wave will be narrow and negative in leads 2, 3, and aVF unlike the wide P waves that result from slow pathway activation (proximal coronary sinus). Once catheters are placed, specific mapping of the fast pathway will show earlier activation than the anatomic slow pathway region (proximal coronary sinus).
5. Activation of the left atrium in sinus rhythm or any right atrial rhythm occurs primarily through Bachmann's bundle and through the coronary sinus musculature. In children, Bachmann's bundle activation can be rapid. This occurrence can give rise to unusual activation patterns in the coronary sinus in children with typical AVNRT (Figure 4). If one follows the circuit of typical AVNRT, antegrade activation is via the posterior input of the AV node (slow pathway), and from the common turnaround point in the AV node, atrial activation breaks through just behind the tendon of Todaro at the fast pathway region. To complete the circuit, activation must now precede from the fast pathway into the slow pathway region either crossing the Eustachian ridge (at times a line of conduction block) or skirting around it between this ridge and the crista terminalis. In children (and some adults), because of rapid conduction over Bachmann's bundle, the circuit may be completed via the left atrial myocardium and electrical connections between the coronary sinus musculature and the left atrium. Thus, the mid-coronary sinus electrograms may be earlier than the proximal coronary sinus. This eccentric activation of the coronary sinus may give the impression of an accessory pathway being present. It should be noted, however, that mapping the fast pathway region (as explained above) will always show earlier activation than the earliest site in the coronary sinus. It should also be noted that eccentric activation when occurring in AVNRT occurs only with typical AVNRT (retrograde fast pathway with left atrial completion of the circuit).

## Wolff-Parkinson-White (WPW) and Related Syndromes - Relevant Anatomy

Wolff-Parkinson-White syndrome results when an accessory pathway is present that electrically connects ventricular and atrial myocardium and gives rise to reentrant supraventricular tachycardia. The electrocardiographic recognition and electrophysiological approach to management of this syndrome in children is discussed elsewhere [10]. Specific causes for difficulty with ablation in children with WPW are discussed in another manuscript in this series. In this paper, I will discuss the anatomy of the variants of this syndrome including unusually located accessory pathways and the anatomical basis for pathways that exhibit unusual electrophysiological behavior.

### Specific anatomic variants

Accessory pathways in children, as well as adults, is sometimes classified into endocardial and epicardial types. In a strict anatomic sense pathways are always epicardial. That is the atrioventricular connection occurs exterior to the fibrous annulus (tricuspid or mitral). However, most of these pathways can be ablated either on the pathway itself or its atrial or ventricular insertion with standard endocardial techniques. In a few exceptional circumstances the pathway is truly epicardial in that the atriovenous connection occurs through another epicardial structure (usually the cardiac veins). In an analogous fashion, pathways that course through the fibrous trigone represent situations where standard endocardial ablation is unlikely to be successful for an anatomic reason.

### Venous pathways

**Figure 5:**
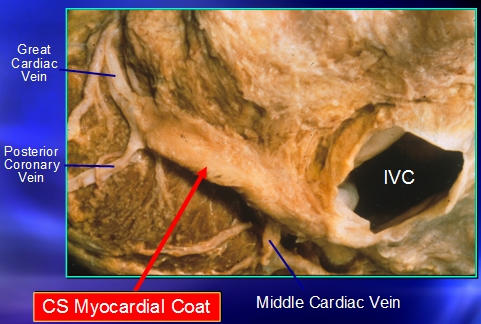
Myocardial extensions exist in various cardiac veins and are consistently seen around the proximal coronary sinus. Note, at the transition of the coronary sinus to the great cardiac vein at the region of the posterior coronary vein, there is an abrupt termination of the myocardial sleeve (see text for details). Courtesy of Dr. Anton Becker, Netherlands.

Muscular extensions (cardiac syncytial) into most cardiac veins including the superior vena cava, pulmonary veins and the coronary sinus exist [11,12]. This myocardial extension into the coronary sinus is electrically continuous at one or more points to the right and left atria (Figure 5). The coronary sinus muscular sleeve usually extends about 4 cm into the vein up to the junction with the great cardiac vein (insertion point of the vein of Marshall and the posterolateral ventricular vein). In some patients this muscular sleeve may extend into one of the ventricular venous branches (middle cardiac vein or posterior veins). In a few individuals this muscular extension into the ventricular veins interdigitates with ventricular myocardium, thus forming a true atrioventricular bypass tract connecting the atrium and ventricle electrically via the muscular sleeves of the coronary sinus and ventricular veins. Since the ventricular veins and coronary sinus are "epicardial" ablation requires a modified approach. Based on understanding the anatomy of these connections either ablation of the ventricular insertion within the middle cardiac vein or circumferential isolation of the ventricular vein (middle cardiac or posterior vein), isolation of the coronary sinus or ablation of the atrial insertions of the coronary venous musculature can be attempted. Each of these approaches has benefits and difficulties that can be readily understood from the underlying anatomy. Ablation at the ventricular insertion site often is deep within the middle cardiac vein and injury to the neighboring posterior descending artery or branch may occur. While isolation of the coronary sinus or disarticulation by ablating each atrial insertion has been done it is a technically very difficult procedure as multiple atrial connections typically exist. When this is accomplished pacing (or a spontaneous rhythm) arising in the coronary vein musculature may still show pre-excitation; however, reentrant tachycardias should no longer be possible. An approach of ablating more proximal in the ventricular vein attempting to isolate that branch alone (middle cardiac vein, diverticulum or posterior vein) is often an anatomic approach taken in these situations.

### Trigone pathways

While technically atrioventricular connections (accessory pathways) should be possible at any point along the atrioventricular annulus, an exception occurs in the region of the aortic mitral continuity. Thus, left anteroseptal accessory pathways (and left anterior) are exceedingly rare compared to other locations. This is because of the unique position of the aortic valve preventing direct atrioventricular connections as could occur across the annulus in other locations. For accessory pathways to occur here, the cardiac muscle (pathway) would have to apparently cross the central fibrous trigone. While this may occur another possibility more evident from recent case description is a connection from the atrial myocardium to the supra-aortic valvar myocardial extension (see below) under ventricular tachycardia and from there to the ventricular myocardium [13]. The situation therefore is analogous to epicardial pathways that involve the cardiac veins. Here again, a non-annular structure with a myocardial sleeve forms part of the course of such pathways. Ablation of such pathways can occur at the atrial or ventricular insertion but is typically best accomplished in the right or noncoronary cusp of the aortic valve. Care must be taken to avoid damage to the AV node (avoid sites with large atrial signal), the aortic valve itself or the right coronary artery.

### Appendage pathways

Another non-annular anatomic situation where atrial and ventricular myocardial tissue is apposed is with respect to the appendages. Accessory pathways that involve electrically active myocardial connection occur between the appendages and the underlying ventricle. Although rare, when seen, these connections constitute an accessory bypass tract as conduction can proceed through these fibers independent of the AV node. When such tracts are diagnosed in children, the optimal approach is to ablate in a circumferential manner isolating a portion of the left or right atrial appendage close to its ostium. While the actual connection may also be targeted deep in the appendage, the risk of perforation in children is high. Targeting the ventricular insertion is typically futile as it is often multiple.

## Anatomy of physiologically distinct accessory pathways

Some accessory pathways may be located in the typical atrioventricular annular location, however, demonstrate unusual physiological properties such as decremental conduction or lack of typical responses to decremental atrial pacing etc. The nomenclature for these pathways is confusing with historical and contemporary usage significantly different [14]. The anatomy of these pathways is briefly reviewed below.

### Mahaim fibers

In present electrophysiology terminology, a Mahaim fiber is a true accessory bypass tract connecting the free wall of the right atrium to either ventricular myocardium or the infra-Hisian conduction system. Unlike a typical accessory pathway, however, Mahaim fibers have AV node-like characteristics near the annulus and connect to relatively apical myocardium or the infra-Hisian right bundle-branch system through an insulated fascicle similar to the His bundle/right bundle-branch.

These pathways, when they occur in children with relatively rapid AV nodal conduction, can be exceedingly difficult to recognize as accessory pathways [15,16]. They are characterized by the absence of retrograde conduction and early ventricular activation during atrial pacing occurring away from the annulus often at an identical site as the right bundle exit via AV nodal conduction. This is because these pathways may directly insert into the right bundle or the moderator band. The absence of retrograde conduction and the absence of dual AV nodal physiology distinguished these structures from true duplication of the AV node, a rare entity. Because these structures typically do not insert into the ventricular myocardium near the annulus mapping the earliest ventricular activation is of minimal utility. A His bundle or Purkinje like potential associated with Mahaim fiber conduction should be targeted, preferably near the annulus for best results. Understanding the anatomy of these tracts will aid the ablation procedure in children complicated by their small heart size and ease with which mechanical trauma (bumping) can lead to transient loss of conduction and prevention of further mapping [17].

### Retrograde Decremental Pathways

While Mahaim fibers do not conduct retrograde, pathways that conduct either exclusively retrograde or bidirectionally occur and are responsible for the syndrome labeled PJRT (permanent form of junctional reciprocating tachycardia). These pathways are distinguished from Mahaim fibers by having the ventricular and atrial insertions close to the annulus and conducting retrograde. There is no relationship anatomically with these pathways and the normal conduction system. For unclear reasons, retrograde decremental pathways are often found in the region of the pyramidal space, close to the coronary sinus ostium in the posterior right or left atrium [18]. Pathways associated with Ebstein's anomaly presumably because of the long course traversing the atrialized ventricular myocardium give rise to the long conduction times and decremental properties.

### Nodoventricular / Nodofascicular Pathways

Unlike a Mahaim fiber that connects atrial myocardium to the ventricular myocardium, nodoventricular pathways connect compact AV nodal tissue either to the fascicular system (right or left bundle branch) or the ventricular myocardium. For these pathways to occur and participate in tachycardia, some form of longitudinal dissociation in the AV node is required, that is antegrade conduction through the AV node and then down the nodoventricular/fascicular tract and then retrograde to AV nodal tissue occurs over a conduction interval too short to be allowed by the usual refractory period of the AV node. The anatomic basis for such dissociation or in fact a convincing example of the anatomy/pathology of these connections is lacking and has prompted some electrophysiologists to question their existence [19,20]. Although the anatomic delineation of these pathways has not been clearly established in the literature, physiologically, some patients have pre-excitation, however, with necessary conduction through the AV node to effect the pre-excitation. Unlike fasciculoventricular tracts (see below), tachycardia can be induced with these arrhythmias and reset from both the ventricle (without entering the compact AV node) or the atrium (only by entering the compact AV node) [21].

### Fasciculoventricular Tracts

Normally, there is a fibrous sleeve of insulation on the penetrating bundle of His and the right and left bundle branches until this sleeve disappears and the bundle branch tissue connects to the ventricular myocardium (bundle branch exit). In some patients, there is a breach in this insulation and ventricular excitation occurs closer to the base on the septum either directly from the His bundle or from the proximal bundle branches. This is termed a fasciculoventricular connection or pathway. The reader should note that these do not represent true atrioventricular accessory bypass tracts since anatomically there is no direct connection from the atrium to ventricular myocardium. Importantly, when the AV node blocks, there is no pre-excitation and progressive decrement in AV nodal conduction is not associated with progressive pre-excitation as seen with typical atrioventricular bypass tracts. The pediatric electrophysiologist should be cognizant of this anatomic variant and differentiate these electrophysiologically from true bypass tracts since fasciculoventricular tracts are not associated with tachycardia and should not be targeted for ablation.

## Atrial Flutter and Atrial Fibrillation

In the pediatric population, in patients without prior cardiac surgery or congenital heart disease, atrial flutter and atrial fibrillation are uncommon. While atrial flutter is one of the most frequent causes of fetal tachycardia, after birth, the arrhythmia is rarely seen without coexisting cardiac disease. Similarly, atrial fibrillation in the pediatric age group is unusual without coexisting disease such as the Holt-Oram syndrome or with familial/inherited atrial fibrillation. From the anatomic perspective, a few important differences are significant with pediatric ablation and are outlined below [22].

### The Cavo-Tricuspid Isthmus

**Figure 6:**
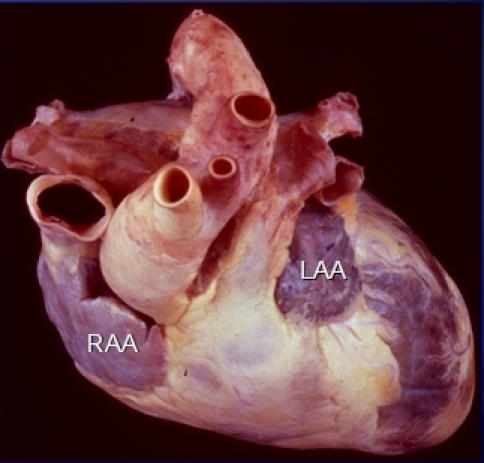
Anatomic dissection showing the complex relationships between the right and left ventricular outflow tracts (Superior view). Note that the ascending aorta is very close to the SVC and is to the right of the patient's body when compared to the right ventricular outflow tract and pulmonary artery. The atrial appendages (RAA and LAA) drape over the outflow tracts with the left ventricular outflow tract and ascending aorta being closer to the RAA and the right ventricular outflow tract and pulmonary artery being in proximity to the LAA.

The cavo-tricuspid isthmus also referred to as the subeustachian isthmus is the critical zone for the typical atrial flutter circuit [23]. This is the region of atrial myocardium between the tricuspid valve and the inferior vena cava including the region of the Eustachian ridge [24]. In infants and young children, the subeustachian isthmus often has a paucity of atrial myocardium and a distinct subeustachian pouch forms. Younger children tend to also have a relatively large thebesian valve [25]. Subeustachian pouches tend to be seen in patients (even in adults) who have a prominent thebesian valve (Figure 6). Thus, with ablation of the subeustachian isthmus in children, care should be given to avoid perforation or coagulum formation in this pouch.

Recognizing the occurrence of this pouch is also important when attempting to cannulate the coronary sinus including for cardiac resynchronization procedures, and this issue if explained more fully in another manuscript in this series.

### Pulmonary Vein Anatomy

When ablating atrial fibrillation in children, the pulmonary veins are typically isolated. However, the veins tend to be smaller in children and the sleeve of myocardium entering the vein is also smaller [26]. Further, conduction velocities tend to be fast and the typical decrement or delay in conduction seen at the pulmonary vein ostium may not be as apparent [27,28]. Great care must be taken to perform all ablation in the atrium (proximal to the ostium). This is because ablation at this site is required to isolate the heterogenous arrhythmogenic tissue formed at the ostia and more importantly to avoid pulmonary vein stenosis. When ablating within the pulmonary vein, endothelial proliferation dominates and stenosis can occur, whereas ablating at sites of more atrial myocardium in addition to causing endothelial proliferation produces aneurysmal dilation of the myocardial layer making stenosis unlikely [29,30].

### Pulmonary Vein Branching

**Figure 7:**
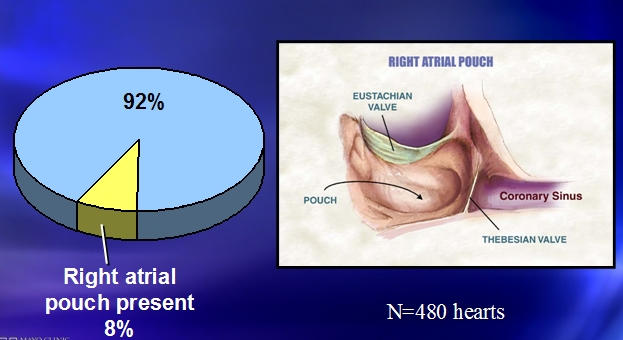
***Prevalence of Right Atrial Pouch:*** Children more commonly, as well as some adults, have a pouch in the subeustachian region frequently associated with a prominent thebesian valve. These pouches may make ablation of typical atrial flutter or cannulation of the coronary sinus challenging in children [25].

Because of the small pulmonary veins and branches, acute occlusion of one of the venous branches may occur during ablation. When venography is performed, it can be difficult to know whether a branch has been occluded or did not exist at all. A simple anatomic rule of pulmonary vein branching can assist the operator in making this decision. Daughter veins that tend to coalesce into a parent trunk have a consistent relationship in terms of the diameter or circumference. The diameter (or circumference) of the daughter veins (tributaries) when added up always is greater (110%) than the diameter (or circumference) of the main vein (Figure 7).

## Ventricular Tachycardia in Children - Relevant Anatomy

Although ventricular tachycardia ablation in children has gone from a rarely performed procedure to more frequently done, it still remains most likely required in children with congenital heart disease or other structural abnormalities. When significant structural abnormality is present (cardiomyopathy, tetralogy of Fallot), a defibrillator has often been placed, and the ablation procedure performed to minimize frequent shocks. The use of defibrillators in children and principles of radiofrequency ablation in pediatric scar-related VT are covered elsewhere in these discussions and this supplement. In this paper, we will expand on the anatomic basis of the two most common ventricular tachyarrhythmias found in children with otherwise structurally normal hearts.

### Outflow Tract Ventricular Tachycardia

Outflow tract ventricular tachycardia is characterized by exercise-related wide QRS tachycardia that mostly occurs in active older boys and less commonly as paroxysmal VT in postmenarchal girls. In a few children with right ventricular outflow tract tachycardia, underlying cardiomyopathy, particularly arrhythmogenic right ventricular cardiomyopathy is found, but the vast majority have no obvious structural heart disease. An accurate understanding of the underlying anatomy of the outflow tracts is critical to safe catheter manipulation, mapping, and ablation of this arrhythmia in children. Pediatric ablationists should familiarize themselves with the relative positions of the right and left ventricular outflow tracts, myocardial sleeves that extend beyond the semilunar valves, and knowledge of the coronary arterial systems relative to both the left and right ventricular outflow tracts.

### "Right" vs. "Left" Outflow Tracts

**Figure 8:**
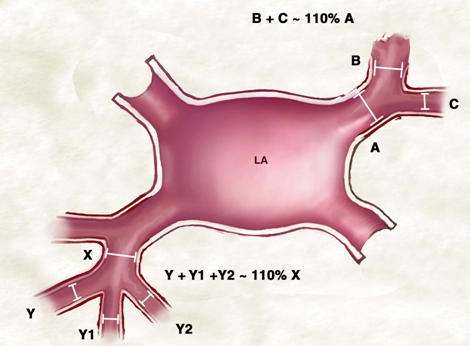
***Relationship of Pulmonary Vein Branches to the Parent Trunk:*** A consistent pattern of pulmonary vein branching is noted. The diameter (or circumference) of the parent trunk is slightly smaller than the diameter (circumference) combined of the branches (see text for details).

The right ventricular outflow tract courses anterior and leftward in the body and at the level of the semilunar valves is to the left of the left ventricular outflow tract. The angle formed between these two outflow tracts varies with age from about 60º in infants to about 90º in older children. Thus, when mapping an outflow tract tachycardia in the right ventricular outflow tract, novice ablationists may consider mapping the left ventricular outflow tract if earlier sites of activation are found as the catheter is positioned more leftward, however, left of the right ventricular outflow tract is some left ventricular myocardium and the mitral annular region anterolaterally. Earlier sites of activation, particularly if the early signal is far-field in nature when located *rightward and posteriorly* should prompt consideration of left ventricular outflow tract mapping. Similarly, when mapping the left ventricular outflow tract, leftward and anterior activation sites should prompt reconsideration of meticulous mapping of the posterior right ventricular outflow (a site where catheter positioning is not straightforward) (Figure 8).

### Myocardial Sleeves Extending Beyond the Semilunar Valves

Myocardial sleeves have been well described that extend beyond the semilunar valves to various lengths endocardial to the great arteries. While these occur in all age groups, the relative lengths of these myocardial sleeves appear to be longer in infants and young children [31]. When mapping the right or left ventricular outflow tracts, it is important to include supravalvar mapping including the regions close to the ostia of the main coronary arteries in the case of the aortic valve [32]. The quality of these signals is different in many cases from the ventricular myocardium because of delay in conduction often seen at the level of the semilunar valves. It should be noted that simply the presence of signals above the semilunar valves is not necessarily arrhythmogenic (in present thinking), and this myocardium may be a bystander that is passively activated from a focus of tachycardia below the valve [33].

### Coronary Arterial Anatomy

Because the pulmonic valve is cephalad and leftward of the aortic valve, the posterior portion of the right ventricular outflow tract just above and at the level of the pulmonic valve is very close to the *left* main coronary artery. While it is common for ablationists to consider performing coronary angiography when ablating in the left ventricular outflow tract, the proximity of the ostium and initial course of the left main coronary artery is closer to the a catheter position to deliver radiofrequency energy in the posterior right ventricular outflow tract close to the pulmonic valve. Because of the relatively shorter angle formed in very young patients and the lack of thick myocardial separation, particular care when ablating at these locations in children is required. When doubt exists, either intracardiac echocardiography to carefully document the separation between the ablating catheter and the coronary arteries (if technically adequate views obtained) or coronary angiography should be performed [34].

### Fascicular Ventricular Tachycardia

Exercise-related ventricular tachycardia in structurally normal hearts that exhibit a right bundle branch block superior access morphology is usually mapped and ablated in the region of the left posterior fascicle (Belhassen's VT). While the heart is typically structurally normal, tissue that traverses the left ventricular cavity from the septum to the free wall is sometimes found close to the region of successful ablation. These have been variously referred to as false tendons, interpapillary muscle chords, left ventricular chords, or lancisi fibers. While such structures are commonly found even in patients without ventricular tachycardia that has been documented when clearly recognized with an imaging modality (echocardiography), their presence can guide the ablationist, particularly when tachycardia has been difficult to induce.

## Summary

An important trend in contemporary electrophysiology is anatomy-based ablation. Although the pediatric electrophysiologist still needs to clearly understand the physiological principles underlying various mapping, diagnostic, and ablation maneuvers, an appreciation of the underlying cardiac anatomy has become critical.

Understanding cardiac anatomy will help minimize complications including collateral damage to the AV conduction system, pulmonary veins, and neighboring structures in the pyramidal space of the heart. Further, an appreciation of the complex anatomy of the ventricular outflow tracts, particularly in children allows accurate correlation between mapping, electrocardiographic, and imaging data. Such understanding again decreases the likelihood of damage to the coronary arteries while facilitating successful ablation.
